# Association of Increased Serum Leptin with Ameliorated Anemia and Malnutrition in Stage 5 Chronic Kidney Disease Patients after Parathyroidectomy

**DOI:** 10.1038/srep27918

**Published:** 2016-06-16

**Authors:** Yao Jiang, Jingjing Zhang, Yanggang Yuan, Xiaoming Zha, Changying Xing, Chong Shen, Zhixiang Shen, Chao Qin, Ming Zeng, Guang Yang, Huijuan Mao, Bo Zhang, Xiangbao Yu, Bin Sun, Chun Ouyang, Xueqiang Xu, Yifei Ge, Jing Wang, Lina Zhang, Chen Cheng, Caixia Yin, Jing Zhang, Huimin Chen, Haoyang Ma, Ningning Wang

**Affiliations:** 1 Department of Nephrology, The First Affiliated Hospital with Nanjing Medical University, Nanjing, Jiangsu Province, 210029, China; 2 Department of General Surgery, The First Affiliated Hospital with Nanjing Medical University, Nanjing, Jiangsu Province, 210029, China; 3 Department of Epidemiology and Biostatistics, School of Public Health, Nanjing Medical University, Nanjing, Jiangsu Province, 210029, China; 4 Department of Nephrology, Jiangsu Province Geriatric Hospital, Nanjing, Jiangsu Province, 210024, China; 5 Department of Urology, The First Affiliated Hospital with Nanjing Medical University, Nanjing, Jiangsu Province, 210029, China

## Abstract

Leptin is an adipokine that regulates various metabolism, but its association with secondary hyperparathyroidism (SHPT), a clinical manifestation of chronic kidney disease-mineral and bone disorder (CKD-MBD), remains obscure. Parathyroidectomy (PTX) is recommended for severe SHPT patients. Here, the associations between circulating leptin and clinical characteristics in CKD patients were investigated. Effects of PTX on leptin production were analyzed *in vivo* and *in vitro*. Controls and CKD patients had approximate serum leptin levels in that a larger proportion of CKD patients with body mass index (BMI) <23 kg/m^2^. Serum leptin was related to anemia, albumin, and bone metabolism disorders in CKD patients. Lower intact parathyroid hormone (PTH) was related with higher leptin in PTX patients group. Severe SHPT inhibited uremia-enhanced leptin production in 3T3-L1 adipocytes, which was attenuated after PTX. High levels of PTH were found to reduce Akt phosphorylation and leptin production *in vitro* but high levels of calcium and phosphorus were not. Successful PTX was found to improve anemia and malnutrition in severe SHPT patients, and this was correlated with increased circulating leptin levels via up-regulated Akt signaling in adipocytes. These findings indicated the therapeutic potential of leptin and related target pathway for improving survival and quality of life in CKD.

Leptin is a 16 kDa protein hormone product of the *obesity (ob*) gene, which is mainly secreted by adipocytes and cleared by the kidneys^1^. Some studies^2^,^3^,^4^ reported elevated levels of circulating leptin in chronic kidney disease (CKD) patients, whereas others demonstrated opposite results^5^. Serum leptin levels in CKD patients still remain controversial^6^,^7^.

Leptin production in adipocytes is influenced by numerous factors^8^,^9^,^10^,^11^, and related with multiple signaling pathways^11^,^12^,^13^. Uremic serum enhanced the secretion of leptin by adipocytes *in vitro*^9^. Secondary hyperparathyroidism (SHPT) is a common complication in CKD patients manifested with mineral and bone disorder (MBD)^14^. To the best of our knowledge, none of the previous studies have specifically addressed the relationship between SHPT and leptin production in CKD patients. The effects of MBD such as high parathyroid hormone (PTH) levels, hypercalcinemia, hyperphosphatemia^14^, and their regulated signaling pathways on leptin production *in vitro* are also indistinct.

Parathyroidectomy (PTX) is recommended to severe SHPT patients refractory to medical treatment^15^, and for reversing anemia, malnutrition and abnormal bone metabolism^16^. Leptin has a wide spectrum of biological activities such as hematopoiesis, neuroendocrine function, energy homeostasis, and bone metabolism^17^,^18^. However, no previous studies have investigated circulating leptin and its correlations with the above disorders in SHPT patients who have undergone PTX.

The purpose of the present study was to evaluate serum leptin levels and its associations with anemia, nutrition and bone metabolism biochemical parameters in stage 5 CKD patients, and investigate the longitudinal changes in these relationships after PTX. Furthermore, we explored the influence of severe SHPT on leptin production and related signaling pathways in 3T3-L1 adipocytes. The results from this study provide a better understanding of leptin physiology, which may help to establish its clinical role and unfold its therapeutic potential in the treatment of CKD-MBD.

## Results

### Baseline Clinical Characteristics of Healthy Controls and Patients with Stage 5 CKD

The basic clinical characteristics and laboratory values of stage 5 CKD patients (n = 209) and healthy volunteers (n = 100) matched for age and gender are shown in Table 1. Stage 5 CKD patients had evident anemia, lipid, and bone mineral disorders that were not detected in controls. The proportion of low body mass index (BMI) (BMI <23 kg/m^2^, a marker of malnutrition^19^,^20^) in CKD patients was markedly higher than in controls, indicating that CKD patients had lower BMI. Serum leptin levels were adjusted by BMI and transformed using natural logarithm (lnleptin/BMI). Lnleptin/BMI was evidently higher in females than in males both in controls and CKD patients.

Compared to No-PTX patients group, the PTX patients group had a greater percentage of hemodialysis (HD) patients, longer dialysis vintage, and a greater prevalence of more severe bone metabolism disorders such as higher ln (intact PTH) (iPTH). The BMI was similar between No-PTX patients and PTX patients. Also, no significant differences were found in the age, gender proportion, BMI and other laboratory values between the follow-up and non-follow-up groups.

### Approximate Lnleptin/BMI between Controls and Patients Is Attributable to A Larger Proportion of Low BMI in CKD Group

The serum leptin levels increased in parallel to BMI both in controls and CKD patients. The association of lnleptin/BMI levels with BMI remained significantly positive in CKD patients. Compared to controls, lnleptin/BMI was lower in CKD patients whose BMI <23 kg/m^2^ and higher in those BMI >23 kg/m^2^ (Figure S1). Overall, CKD patients showed no significant difference in lnleptin/BMI compared with controls because the proportion of low BMI in CKD patients was greater than in controls. The PTX patients group had lower lnleptin/BMI than the No-PTX patients group, although the difference was not statistically significant.

### Lnleptin/BMI Is Related to Anemia, Albumin, Lipid, and Bone Metabolism in CKD Patients and Controls

The correlations between basic clinical characteristics and serum leptin are shown in Table 2. Serum lnleptin/BMI correlated negatively with renal function parameters in controls. Higher lnleptin/BMI in the No-PTX patients group was related with milder anemia. Lipid biochemical parameters [except high density lipoprotein (HDL) cholesterol)] and albumin (Alb) were both positively related with lnleptin/BMI in No-PTX patients group and PTX patients group. In PTX patients group, higher lnleptin/BMI was correlated with milder MBD such as lniPTH (r = −0.306, *P* = 0.021). However, this was not statistically significant in the No-PTX patients group.

### Postoperative Improvement in Anemia and Malnutrition Was Related to Increased Lnleptin/BMI in Successful PTX Patients

As shown in Table 3, postoperative anemia, BMI, total cholesterol (TC), and MBD were improved in 36 successful PTX patients. Furthermore, postoperative lnleptin/BMI were increased in successful PTX patients (6.0 ± 1.3 *vs* 5.5 ± 1.3). Persistent SHPT patients also had elevated lnleptin/BMI after PTX. However, this was not statistically significant. Patients with low BMI in the successful PTX group demonstrated an increase in their body weight (Table S1). Lnleptin/BMI change percent was positively associated with hemoglobin (Hb) change percent (r = 0.381, *P* = 0.026) and Alb change percent (r = 0.400, *P* = 0.019) in successful PTX group. No time trends were observed in body weight changes, BMI and laboratory values except serum Alb and phosphorus (P) level in the successful PTX group.

### Severe SHPT Inhibited Uremia-enhanced Leptin Production in 3T3-L1 Adipocytes

**Figure 1:**
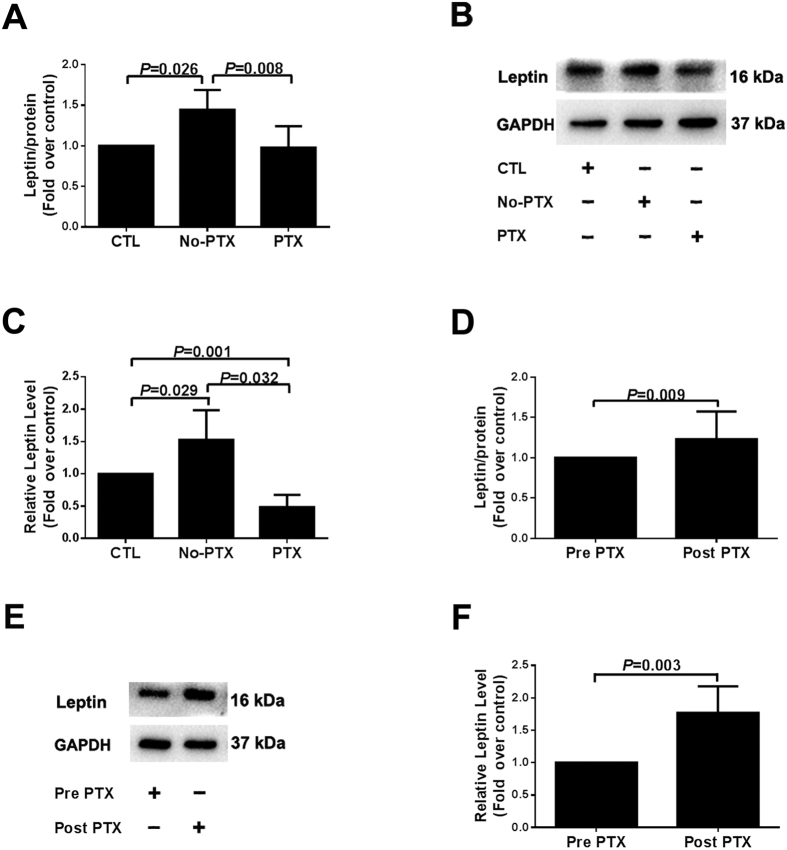
Effects of different human serum on leptin production in 3T3-L1 adipocytes. The differentiated adipocytes were treated with 10% human serum media for 24 h. The serum-stimulated groups included healthy controls (CTL), No-PTX patients (No-PTX), preoperative (PTX or Pre PTX), and follow-up postoperative PTX patients (Post PTX) (n = 5). Leptin production was measured using ELISA or Western blot analysis. (**A,D**) Leptin levels in medium assessed by ELISA. (**B,E**) Leptin protein in cytoplasm detected by immunoblotting. (**C,F**) Densitometry analysis of immunoblotting images. The data from all the groups were normalized corresponding to the control at each time of exposure, respectively. Results were shown as mean ± SD, and error bars were pooled from at least three independent experiments.

3T3-L1 adipocytes were treated with human serum from different CKD patients, and analyzed *in vitro* to verify the effect of severe SHPT on leptin production (Fig. 1). Leptin secretion in the serum of No-PTX CKD patients (No-PTX group) was higher compared with controls. However, leptin production in serum of PTX patients (PTX group) had no significant difference with controls, and was lower than the No-PTX group *in vitro* (Fig. 1A). Similar results were obtained in the leptin expression study using Western blotting (Fig. 1B,C). The results indicated that uremic serum can stimulate more leptin synthesis and secretion in adipocytes; severe SHPT inhibited this increased leptin production.

### Inhibition Effect of SHPT on Leptin Production Was Attenuated in 3T3-L1 Adipocytes after PTX

Clinical data indicated that the levels of serum leptin were greatly increased in severe SHPT patients after PTX. We further investigated whether this increase was secondary to altered leptin production in adipocytes. Interestingly, compared with Pre PTX group (stimulated with preoperative serum), 3T3-L1 adipocytes cultured in postoperative serum (Post PTX group) secreted more leptin into the culture media (Fig. 1D) and stored more leptin in the cytoplasm (Fig. 1E,F). Taken together, these results demonstrated that the inhibited leptin production by severe SHPT was weakened after PTX.

### High PTH, not Calcium or Phosphorus, Reduced Leptin Production *in vitro*

**Figure 2:**
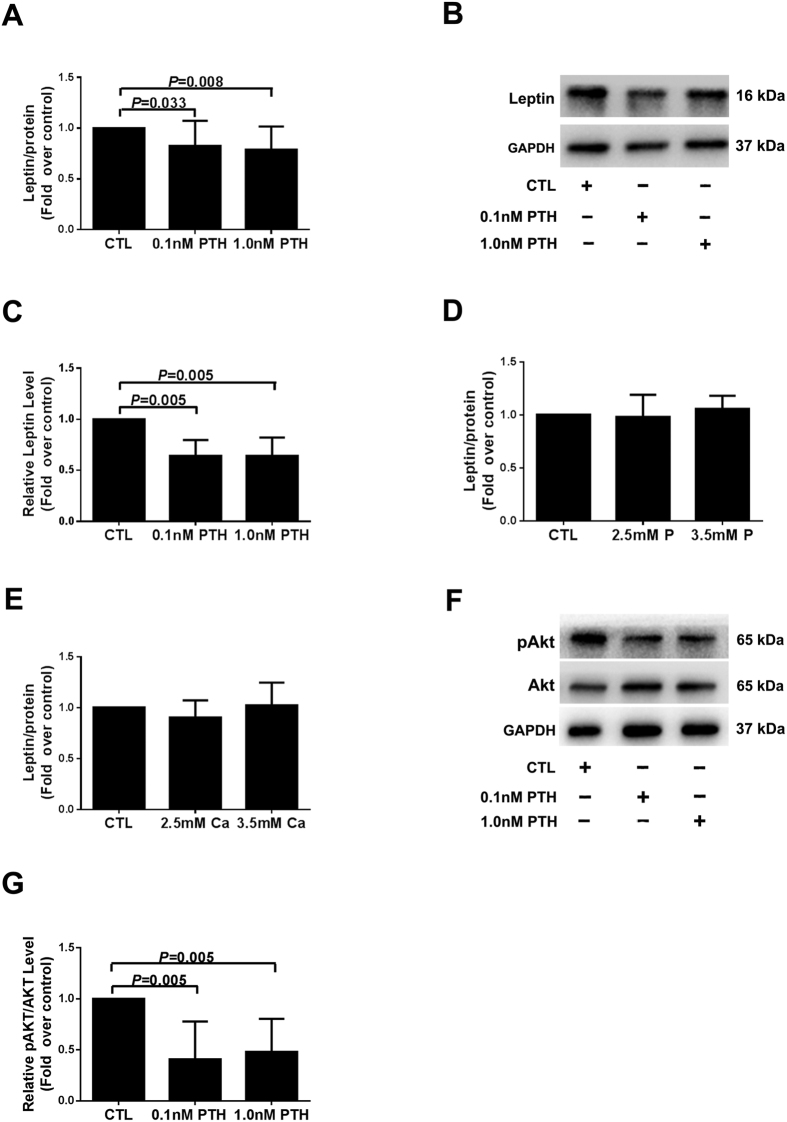
Effects of high PTH, Ca or P on leptin production and Akt phosphorylation in 3T3-L1 adipocytes. The differentiated adipocytes were treated with high levels of PTH (0.1 nM, 1.0 nM), high Ca (2.5 mM, 3.5 mM) or high P (2.5 mM, 3.5 mM) for 24 h. Leptin production was measured using ELISA or Western blot analysis. Akt phosphorylation and Akt expressions were detected using Western blot analysis. (**A,D,E**) Leptin levels in medium assessed using ELISA. (**B,F**) Leptin protein detected by immunoblotting. (**C,G**) Densitometry analysis of immunoblotting images. The data from all these groups were normalized corresponding to the control at each time of exposure. Results were shown as mean ± SD, and error bars were pooled from at least three independent experiments. CTL, controls; Ca, calcium; P, phosphorus; PTH, parathyroid hormone.

It is well known that PTX can correct bone mineral disorders classified as hypercalcemia, hyperphosphatemia and high serum PTH levels in SHPT patients^21^,^22^. Therefore, to investigate the factors contributing to lower circulating leptin levels in severe SHPT, we studied the effects of high PTH, calcium (Ca) or P environment on leptin synthesis and secretion *in vitro*. Compared to controls, stimulation of adipocytes with high PTH (0.1 nM, 1.0 nM) reduced the levels of leptin in both cell culture media (Fig. 2A) and protein extracts (Fig. 2B,C). However, leptin production in adipocytes was not affected by high concentrations of Ca or P (2.5 mM, 3.5 mM) (Fig. 2D,E). Therefore, we speculated that high PTH was a main factor responsible for lower circulating leptin levels in severe SHPT patients.

### High PTH Inhibited both Akt Phosphorylation and Leptin Production in 3T3-L1 Adipocytes

It has been previously reported that PI3K/Akt signaling pathway is an independent mechanism for leptin release^12^. Moreover, PTH can affect the Akt signaling in differentiated 3T3-L1 adipocytes^23^,^24^,^25^. Based on these studies, we focused on the role of Akt signaling in leptin production with high PTH stimulation. We found that both 0.1 nM and 1.0 nM PTH inhibited the phosphorylation of Akt in adipocytes (Fig. 2F,G), indicating that elevated PTH downregulated Akt signaling in adipocytes.

### Up-regulated Akt Signaling Mediated Increased Leptin Production after PTX *in vitro*

**Figure 3:**
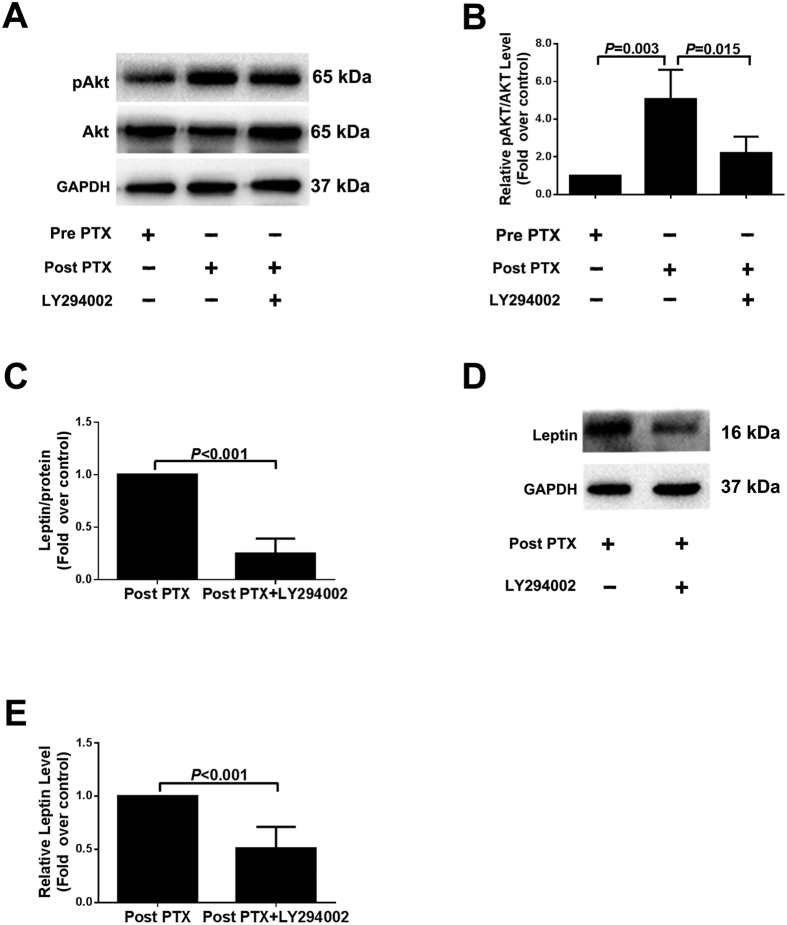
Up-regulated Akt signaling mediated increased leptin production after PTX *in vitro*. The differentiated adipocytes were pretreated with LY294002 (30 μM) for 30 min, and then co-stimulated with 10% preoperative (Pre PTX) or postoperative serum (Post PTX) for another 24 h. Leptin in media was measured using ELISA and leptin in cytoplasm was measured using Western blot analysis. Akt phosphorylation and expressions were detected by immunoblotting. (**A**) Akt phosphorylation and total Akt protein in cytoplasm detected by immunoblotting. (**B,E**) Densitometry analysis of immunoblotting images. (**C**) Leptin level in medium assessed using ELISA. (**D**) Leptin protein in cytoplasm detected by immunoblotting. The data from all the groups were normalized corresponding to the control at each time of exposure, respectively. Data were shown as mean ± SD, and error bars were pooled from at least three independent experiments.

Our clinical data indicated that postoperative serum PTH levels were decreased and leptin levels were increased in severe SHPT patients. We further investigated the effects of PTX on Akt phosphorylation and leptin production *in vitro.* The protein levels of Akt phosphorylation in Post PTX group were increased notably compared to Pre PTX group. However, when the adipocytes were pretreated with LY294002 (Akt signaling pathway inhibitor), both Akt signaling and leptin production were decreased immediately (Fig. 3A,B). Moreover, leptin secretion in Post PTX group also was reduced greatly after pretreatment with LY294002 *in vitro* (Fig. 3C). Similar alterations were seen in leptin expression (Fig. 3D,E), indicating that up-regulated leptin production in Post PTX group was blocked by Akt inhibitor. Overall, these results suggested that elevated leptin production in severe SHPT after PTX was at least partly, secondary to the reduction in circulating PTH, and mediated via up-regulated Akt signaling.

## Discussion

Leptin, an adipokine that is produced in subcutaneous and visceral adipose tissue, is a plausible biological mediator. Its physiological roles include signaling long-term caloric intake and fat stores to the hypothalamus, thereby modifying food consumption and energy expenditure^18^. In CKD patients, leptin plays an important role in hematopoiesis, nutrition and bone metabolism^26^. SHPT, a familiar clinical manifestation of CKD with high mortality, is used to describe a broader clinical syndrome, including mineral, bone and calcific cardiovascular abnormalities^15^. PTX is widely used as a preliminary therapy for drug-resistant SHPT patients to reverse the above disorders^16^,^21^. However, the relationship between leptin and SHPT is poorly known.

In line with numerous studies^3^,^6^,^27^, we found gender differences in the levels of leptin, which was significantly higher in women. Some studies^2^,^3^,^4^ suggested that elevated serum or plasma leptin was due to reduced renal clearance in CKD patients, whereas the opposite view was proposed that decreased clearance by the kidneys did not contribute to elevated leptin levels^6^. Results showed that no differences were observed in circulating leptin levels between CKD patients and healthy controls^5^. In the present study, we demonstrated that lnleptin/BMI in CKD patients was similar to that in controls. The exact cause for discrepancies in circulating leptin levels in CKD patients is unclear and several mechanisms may have been included. First, uremic serum stimulated more leptin release from adipose tissue than controls, although it was not induced by the accumulation of urea^9^. Second, leptin gene expression in uremic adipose tissue was suppressed because of the feedback inhibition of *ob* gene expression induced by hyperleptinemia^28^,^29^. Third, leptin was positively related with BMI whatever in CKD patients or controls. Because a larger proportion of CKD patients in this study had low BMI, approximate serum leptin levels of controls and CKD patients were determined. Fourth, high-flux HD and hemodiafiltration have been shown to reduce circulating leptin levels in HD patients^30^,^31^. In the present study, most patients received high-flux HD.

In our study, hemoglobin in No-PTX patients was positively related with lnleptin/BMI. However, this relationship did not exist in PTX patients because of blood transfusion for correcting anemia before operations. Hyperleptinemia has been shown to be a stimulating factor for erythropoiesis, and that it reflects a better recombinant human erythropoietin (EPO) response in long-term HD patients^32^,^33^. Thus serum leptin levels may be a predictor of EPO sensitivity. Anemia in CKD is a multifactorial process, associated with relative EPO deficiency and dialysis adequacy^34^,^35^. In our research, dialysis modes for PTX patients were unchanged after operations. For this reason, dialysis adequacy might not be a confounder of analysis of anemia in this study. Our results revealed that anemia was evidently improved in postoperative PTX patients, which was related with elevated lnleptin/BMI. It has been shown that bacterial recombinant leptin acts synergistically with EPO to stimulate end-stage colony-forming-unit erythroid development in humans^36^. These findings raise the possibility of leptin supplementation in protection against renal anemia. Previous studies^37^,^38^ have also shown that PTX could improve anemia in SHPT and reduce the EPO dosages. The relationship between the reduced EPO dosage and increased serum leptin level in SHPT patients after PTX could be evaluated further in the future.

In healthy persons, leptin regulates appetite, food intake, and energy expenditure^18^. Clinical studies found conflicting results regarding the relationship between nutritional status and plasma leptin levels in uremic patients^39^. Several studies have demonstrated that increased leptin concentration is associated with anorexia and muscle mass loss. Some studies^40^,^41^ did not find any correlation between leptin concentration and nutritional status of uremic patients. However, low serum leptin levels predicted mortality in HD patients^42^. Elevated serum leptin levels were associated with good nutritional status in non-obese chronic HD patients^43^. In the present study we found a significant negative correlation of serum Alb, a nutrition marker, with serum lnleptin/BMI in controls. A positive trend between lnleptin/BMI and serum Alb levels was also observed in the CKD patients, indicating that patients with higher baseline leptin had better nutritional status. After PTX, serum albumin levels were improved greatly, and related with increased lnleptin/BMI in severe SHPT patients. We hypothesize that complete or relative leptin deficiency is a predictor of malnutrition in CKD, and leptin replacement may be a rational therapeutic option.

It has been previously reported that serum leptin levels are not correlated with total low density lipoprotein (LDL) cholesterol, HDL cholesterol, TC, or TG levels in general population, whereas some studies suggested that serum leptin levels were positively correlated with the above parameters in dialysis patients. We found that the serum lnleptin/BMI levels had an active effect on lipid metabolism in CKD patients. After PTX, most lipid biochemical parameters in SHPT patients were increased, TC/HDL cholesterol, which predicts cardiovascular risk^44^,^45^, did not change significantly. These results indicate that correction of above lipid metabolism abnormalities after PTX in severe SHPT patients does not increase the risk of cardiovascular disease. Long-term leptin administration has a sustained effect to improve dyslipidemia in hypoleptinemic group of lipodystrophic patients^46^. It is promising that leptin would provide favorable outcomes in CKD patients with dyslipidemia.

As the uremic milieu is complex and contains many confounding factors, clinical studies have suggested that circulating leptin levels are affected by several metabolic disorders such as metabolic acidosis, inflammation and insulin resistance^43^,^47^,^48^,^49^. These findings are consistent with basic research that leptin production in 3T3-L1 adipocytes is related with multiple pathways^11^,^12^,^13^. In the present study, we demonstrated that severe SHPT inhibited leptin production compared with the uremic environment, and we further explored the feasible regulatory factors *in vitro*.

Our data revealed that high PTH, not high Ca and P, reduced leptin production by inhibiting Akt phosphorylation in adipocytes, which is consistent with our epidemiological observation of an inverse association of baseline leptin concentrations with serum PTH levels in severe SHPT patients. Other studies have also demonstrated that PTH affects the Akt signaling in adipocytes^23^,^24^,^25^, which is an independent pathway in leptin release^12^. Moreover, we also demonstrated that increased leptin production in severe SHPT patients after PTX was related with up-regulated Akt phosphorylation levels *in vitro*. It is well known that, the key characteristic of SHPT is the elevation of serum PTH levels, which decreases after PTX. Therefore, we speculate that PTH is an important link for leptin production in SHPT.

There are several limitations to our present investigation. First, to speculate the body fat mass for adjusting leptin, BMI might be less accurate than others such as waist circumference^50^. Second, the effects of leptin on persistent SHPT patients were difficult to conclude because of small sample size. Third, the patients we enrolled were from a single center. Further studies with a larger sample size and multicentric samples are needed. Fourth, studies assessing the therapeutic potential of leptin in CKD patients were not performed. Fifth, given that low BMI (BMI <23 kg/m^2^) is considered a marker of malnutrition, we hypothesized that leptin levels would be an important predictor of CKD malnutrition. However, it’s crucial to note that BMI is not the only indicator of malnutrition. Other clinical parameters such as the Medical Outcomes Study Short Form 36-Item Health Survey (SF36) score or subjective global assessment (SGA) score are suitable for inclusion in the assessment of nutritional status in CKD patients^51^,^52^. We are short of the preliminary survey results such as physical examination history and health self-assessment. We will improve our work in the future.

**Figure 4:**
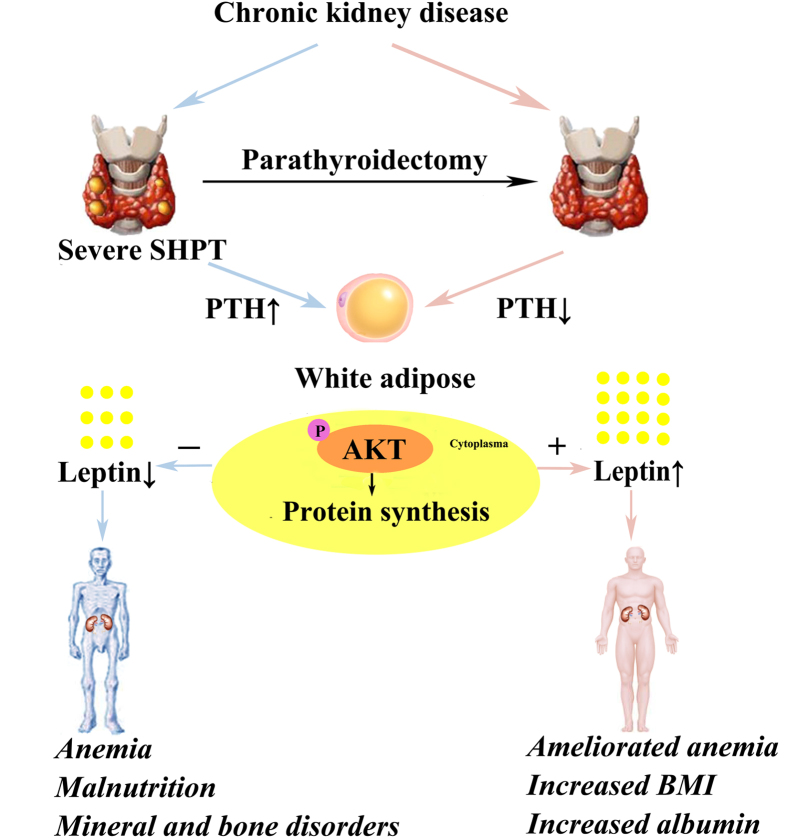
Leptin in CKD: A Link between anemia, malnutrition, and SHPT. SHPT, a common complication of CKD, manifests primarily as high circulating PTH levels, which can inhibit Akt activation and leptin production in adipose. Leptin regulates hematopoiesis and nutrition metabolism. In patients with severe SHPT, after PTX, adipocytes produced more leptin through up-regulated Akt signaling because of normalization with high PTH environment, which in turn increased circulating leptin levels, and finally improved anemia and malnutrition. SHPT, secondary hyperparathyroidism; PTH, parathyroid hormone.

In conclusion, serum leptin in CKD patients was closely related with BMI, and had gender differences with higher levels in women. A larger proportion of patients with BMI less than 23 kg/m^2^ in CKD may contribute to approximate circulating leptin levels in healthy controls. We demonstrated a strong and independent association between lower circulating leptin levels and low BMI, anemia, and reduced albumin levels in CKD patients. We first revealed that adipocytes produced more leptin through up-regulated Akt signaling because of normalization with high PTH environment, which in turn increased circulating leptin levels, and finally improved anemia and malnutrition in severe SHPT patients after PTX. Low BMI could be a marker of malnutrition in CKD patients^19^,^20^. It adversely affects patients’ survival and quality of life. Our findings confirmed that circulating leptin levels may serve as one of the biomarkers for anemia and malnutrition, and more importantly, open new pathways for possible preventive and therapeutic intervention in CKD patients. A model of these relationships is shown in Fig. 4. The use of leptin mimetics and antagonists or targeting leptin signaling may substantially improve the quality of life and survival in CKD patients. Further exploration of the molecular and cellular basis for the observed leptin association may expand our understanding of the pathophysiology and development of CKD-MBD.

## Methods

### Patients

Here, 209 inpatients aged 18–75 years who were treated at the center from March 2011 to April 2015 were enrolled. Patients had an estimated glomerular filtration rate (eGFR) <15 ml/min per 1.73 m^2^ without dialysis or undergoing maintenance dialysis (either peritoneal dialysis or HD). HD treatment was performed for 12 h weekly using bicarbonate dialysate. Peritoneal dialysis was performed every day using glucose-lactate-based peritoneal dialysates. All dialysis patients reached dry weight. The use of EPO followed the Kidney Disease: Improving Global Outcomes (KDIGO) guideline for anemia in CKD patients^53^, EPO was initially used when the Hb concentration of CKD patients fall below 90 g/L and adapted due to the individual response. Lipid management was performed in accordance with the KDIGO guideline^54^. Patients undergoing dialysis were not given statins or statin/ezetimibe combination unless they were already receiving above medication.

Fifty-seven severe SHPT patients (persistent serum levels of iPTH >800 pg/ml[88.0 pmol/l] and confirmed refractory to medical therapy) who underwent total PTX with forearm autotransplantation were enrolled. The PTX patients were recruited from a wide geographical area, some of them dropped out after the operation because of inability to contact the research team, transfer to other dialysis units, poor compliance or death. Forty patients were successfully followed up (median interval was 5.7 months). The relevant clinical definitions of successful PTX, persistent SHPT, and anemia were given in supplemental information.

In this study, none of the patients took calcimimetics. Patients were excluded if they had a past history of PTX or kidney transplant. Participants who were pregnant or had fever, any infection, fasting blood glucose (Glu) on the day of evaluation greater than 200 mg/dl or other problems limiting their normal daily activities were also excluded because they may experience decreased nerve conduction velocity, and this might distort the results of autonomic testing^55^. None of individuals had suffered from any malignant tumors, acute myocardial infarction^56^, liver cirrhosis and severe mental disorders^57^. Those treated with immunosuppressive drugs, calcitonin or bisphosphonates were also excluded. In addition, no participants had morbid obesity (BMI >40 kg/m^2^) or any other serious medical problems.

### Control Group

To compare with the stage 5 CKD patients, we enlisted 100 healthy volunteers matched for age and gender. Exclusion criterions were the same as the patients studied, and included any known renal diseases.

### Collection of Human Serum Samples

In our study at enrollment, venous whole blood samples were drawn in the morning from the participants with an overnight fast. For hemodialysis patients, blood samples were collected before dialysis. Serum samples were analyzed as described in supplemental experimental procedures. Serum Leptin levels were determined using Human Leptin ELISA kits. All patients and controls gave written informed consent, and the study protocols were approved by the Research Ethics Committee of The First Affiliated Hospital with Nanjing Medical University, People’s Republic of China. All clinical investigations were conducted in accordance with the 2008 Helsinki Declaration and good clinical practice guidelines.

### Cell Culture

Mouse 3T3-L1 pre-adipocytes (Chinese Academy of Medical Sciences) were cultured and differentiated toward adipocytes as described previously. Cells were starved for 24 hours and then stimulated with different types of 10% human serum or different concentrations of P, Ca, and PTH. The serum stimulated groups included healthy controls, No-PTX CKD patients, preoperative and follow-up postoperative PTX patients (n = 5). In Post PTX group, cells were pretreated with LY294002 (30 μM) for 30 minutes. Medium and cell protein were harvested after 24 hours. Leptin concentrations in the medium were measured by ELISA kits. Leptin, phosphate-Akt and Akt protein in cytoplasm were detected using Western blot analysis. This was described in the supplemental information. See supplemental experimental procedures for further details about the origins of the reagents. Experiments were repeated thrice. Bars in the figures represent the relative ratio of control group.

### Statistical Analysis

Normality of all the variables was tested by the One-Sample Kolmogorov-Smirnov Test. Variables showing a positively skewed distribution were natural log-transformed. Comparisons were performed using independent samples *t* test, Wilcoxon rank sum test, chi-squared, Fisher’s Exact Test, a paired sample *t* test or ANOVA. The correlation coefficients were calculated by Pearson’s correlation. Differences were considered to be significant when the probability value was <0.05. Statistical analyses were performed with Statistical Package for the Social Sciences (SPSS) version 20.0 (SPSS Inc., Chicago, IL) for Windows software.

## Additional Information

**How to cite this article**: Jiang, Y. *et al*. Association of Increased Serum Leptin with Ameliorated Anemia and Malnutrition in Stage 5 Chronic Kidney Disease Patients after Parathyroidectomy. *Sci. Rep.* **6**, 27918; doi: 10.1038/srep27918 (2016).

## Supplementary Material

Supplementary Information

## Figures and Tables

**Table 1:** Clinical characteristics and baseline laboratory results of study groups.

**Variables**	Controls(n = 100)	Stage 5 CKDPatients(n = 209)	***P***	No-PTX Patients Group(n = 152)	**PTX Patients Group**				*P*^***′***^
					Non-follow-up(n = 17)	Follow-up (n*** = *****40)**		Total (n = 57)	
						Successful PTX(n = 36)	Persistent SHPT(n = 4)		
Demographics									
Age (years)	49.3 ± 13.3	49.4 ± 13.2	0.937	50.8 ± 13.9	44.3 ± 10.4	46.9 ± 10.5	38.5 ± 4.8	45.5 ± 10.3	0.003
Male/Female	47/53	111/98	0.315	78/74	10/7	19/17	4/0	33/24	0.396
BMI (kg/m^2^)	23.7 ± 2.8	21.7 ± 3.2	<0.001	21.9 ± 3.2	20.8 ± 3.5	21.5 ± 3.1	19.6 ± 2.5	21.2 ± 3.2	0.128
BMI <23(kg/m^2^), n (%)	40(40.0%)	135(64.6%)	<0.001	98(64.5%)	11(64.7%)	23(63.9%)	3(75.0%)	37(64.9%)	0.953
Systolic BP (mmHg)	123.0 ± 16.0	146.3 ± 26.4	<0.001	150.2 ± 26.4	128.3 ± 31.4	138.1 ± 18.8	151.3 ± 24.6	136.1 ± 23.9	0.001
Diastolic BP (mmHg)	78.1 ± 10.8	88.0 ± 15.4	<0.001	89.3 ± 15.0	80.5 ± 22.7	86.0 ± 12.4	91.3 ± 10.3	84.7 ± 16.1	0.057
Dialysis mode, n (%)									
Predialysis	100 (100.0%)	57 (27.3%)	<0.001	57 (37.5%)	0 (0.0%)	0 (0.0%)	0 (0.0%)	0 (0.0%)	<0.001
Haemodialysis	0 (0.0%)	118 (56.5%)	<0.001	63 (41.4%)	16 (94.1%)	36 (100.0%)	3 (75.0%)	55 (96.5%)	<0.001
Peritoneal dialysis	0 (0.0%)	34 (16.3%)	<0.001	32 (21.1%)	1 (5.9%)	0 (0.0%)	1 (25.0%)	2 (3.5%)	0.002
Dialysis vintage (months)	NA	48.0 (9.0–96.0)	NA	12.0 (5.0–49.5)	84.0 (61.5–115.5)	84.0 (60.0–120.0)	97.0 (69.0–132.5)	84.0 (60.0–120.0)	<0.001
Comorbidities, n (%)									
Diabetic Mellitus	0 (0.0%)	33 (15.8%)	<0.001	31 (20.4%)	1 (5.9%)	1 (2.8%)	0 (0.0%)	2 (3.5%)	0.003
Hypertension	11 (11.0%)	171 (81.8%)	<0.001	130 (85.5%)	9 (52.9%)	29 (80.6%)	3 (75.0%)	41 (71.9%)	0.023
Cause of ESRD, n (%)									
Glomerulonephritis	0 (0.0%)	152 (72.7%)	<0.001	99 (65.1%)	15 (88.2%)	34 (94.4%)	4 (100.0%)	53 (93.0%)	<0.001
Diabetic nephropathy	0 (0.0%)	20 (9.6%)	0.001	20 (13.2%)	0 (0.0%)	0 (0.0%)	0 (0.0%)	0 (0.0%)	0.004
Hypertensive nephropathy	0 (0.0%)	7 (3.3%)	0.101	7 (4.6%)	0 (0.0%)	0 (0.0%)	0 (0.0%)	0 (0.0%)	0.193
Polycystic kidney disease	0 (0.0%)	13 (6.2%)	0.012	11 (7.2%)	1 (5.9%)	1 (2.8%)	0 (0.0%)	2 (3.5%)	0.521
Other	0 (0.0%)	17 (8.1%)	0.003	15 (9.9%)	1 (5.9%)	1 (2.8%)	0 (0.0%)	2 (3.5%)	0.164
Anti-hypertension Medication, n (%)									
Calcium channel blocker	1 (1.0%)	124 (59.3%)	<0.001	96 (63.2%)	5 (29.4%)	21 (58.3%)	2 (50.0%)	28 (49.1%)	0.066
ACEI/ARB	0 (0.0%)	41 (19.6%)	<0.001	29 (19.1%)	2 (11.8%)	8 (22.2%)	2 (50.0%)	12 (21.1%)	0.749
beta-Receptor blocker	0 (0.0%)	72 (34.4%)	<0.001	50 (32.9%)	7 (41.2%)	14 (38.9%)	1 (25.0%)	22 (38.6%)	0.440
Laboratory values									
Hemoglobin (g/l)	144.1 ± 15.5	92.0 ± 22.9	<0.001	89.2 ± 22.0	103.4 ± 31.8	96.6 ± 19.5	109.0 ± 17.2	99.5 ± 23.6	0.003
Hematocrit (%)	43.2 ± 4.3	28.3 ± 7.1	<0.001	27.3 ± 6.7	32.1 ± 10.3	30.1 ± 5.9	34.1 ± 5.0	31.0 ± 7.4	0.001
Glucose (mmol/l)	5.4 ± 0.8	4.9 ± 1.9	0.002	5.2 ± 2.1	4.4 ± 0.9	4.3 ± 1.3	4.0 ± 0.6	4.3 ± 1.1	<0.001
Creatinine (μmol/l)	71.3 ± 15.8	879.8 ± 351.5	<0.001	887.9 ± 387.4	827.3 ± 207.7	854.6 ± 232.7	1024.0 ± 310.1	858.3 ± 231.5	0.502
Urea (mmol/l)	5.5 ± 1.4	25.1 ± 9.9	<0.001	26.1 ± 10.6	20.7 ± 5.3	23.0 ± 7.6	25.5 ± 7.2	22.5 ± 7.0	0.005
HDL cholesterol (mmol/l)	1.4 ± 0.3	1.1 ± 0.3	<0.001	1.1 ± 0.3	1.1 ± 0.4	1.0 ± 0.3	1.1 ± 0.2	1.1 ± 0.3	0.775
LDL cholesterol (mmol/l)	2.8 ± 0.7	2.8 ± 0.9	0.733	2.9 ± 0.9	2.4 ± 0.6	2.5 ± 0.6	2.7 ± 0.5	2.5 ± 0.6	<0.001
TC (mmol/l)	5.0 ± 0.8	4.4 ± 1.2	<0.001	4.6 ± 1.2	4.1 ± 1.0	3.9 ± 0.8	4.5 ± 1.1	4.0 ± 0.9	<0.001
Triglyceride (mmol/l)	1.4 ± 1.4	1.6 ± 1.2	0.335	1.6 ± 1.2	1.4 ± 0.8	1.5 ± 0.9	1.9 ± 1.3	1.5 ± 0.9	0.487
TC/HDL cholesterol	3.7 ± 0.9	4.3 ± 1.4	<0.001	4.5 ± 1.4	3.7 ± 0.7	4.0 ± 1.2	4.4 ± 1.2	3.9 ± 1.1	0.004
Albumin (g/l)	47.6 ± 2.9	36.8 ± 4.9	<0.001	36.5 ± 5.2	38.7 ± 5.6	37.4 ± 3.3	37.0 ± 3.3	37.7 ± 4.1	0.069
Leptin (pg/ml)	5264.1(2782.0–8865.1)	5221.8(1639.4–13756.3)	0.741	4832.1(1590.0–13841.5)	5524.4(1878.1–21711.0)	5979.0(1989.1–12710.1)	1189.3(288.4–2650.5)	5524.4(1673.2–13712.3)	0.855
Male	2778.2(2072.3–4522.6)	2908.7(1170.7–6691.0)	0.809	2924.2(1324.3–7045.1)	2858.0(1292.8–7556.1)	3027.5(824.8–7931.8)	1189.3(288.4–2650.5)	2565.0(856.7–6514.3)	0.413
Female	7906.3(5553.0–10887.7)	9876.7(3352.5–26268.9)	0.258	9121.1(2595.9–27426.6)	23720.8(5524.4–27640.4)	9983.2(5752.3–19948.2)	NA	10660.0(5652.2–23932.8)	0.558
Leptin/BMI	218.5(119.3–393.9)	242.7(84.4–671.5)	0.781	232.0(80.9–687.2)	269.7(84.0–860.2)	281.4(100.0–541.9)	64.0(16.0–117.5)	259.4(86.3–572.0)	0.939
Male	118.8(85.6–187.0)	135.2(56.5–298.7)	0.614	135.3(63.2–329.6)	157.6(70.2–312.9)	149.9(40.2–287.9)	64.0(16.0–117.4)	129.3(44.7–272.4)	0.548
Female	351.5(273.1–482.9)	464.0(169.2–1152.3)	0.124	419.4(123.2–1158.9)	881.1(353.6–1452.4)	492.0(273.2–1040.5)	NA	536.2 (296.3–1046.4)	0.558
ln Leptin/BMI	5.4 ± 0.7	5.4 ± 1.4	0.557	5.5 ± 1.4	5.6 ± 1.3	5.5 ± 1.3	3.8 ± 1.3	5.4 ± 1.3	0.761
Male	4.8 ± 0.6	4.9 ± 1.1	0.521	5.0 ± 1.1	5.0 ± 1.0	4.9 ± 1.2	3.8 ± 1.3	4.8 ± 1.2	0.460
Female	5.8 ± 0.5	6.0 ± 1.4	0.236	6.0 ± 1.5	6.4 ± 1.2	6.1 ± 1.0	NA	6.2 ± 1.0	0.381
Bone metabolism panel									
Calcium (mg/dl)	9.4 ± 0.4	9.1 ± 1.3	0.002	8.7 ± 1.2	10.2 ± 1.0	10.1 ± 1.2	9.5 ± 0.9	10.1 ± 1.1	<0.001
Phosphorus (mg/dl)	3.7 ± 0.5	6.6 ± 2.0	<0.001	6.6 ± 2.1	6.4 ± 1.2	7.0 ± 2.2	7.0 ± 0.9	6.8 ± 1.9	0.384
ALP (u/l)	74.7(63.3–86.9)	97.4(72.7–206.9)	<0.001	82.0(66.0–107.8)	300.8(184.3–544.3)	552.7(294.8–969.1)	852.5(574.8–1107.1)	506.3(286.1–951.6)	<0.001
lnALP	4.3 ± 0.3	4.9 ± 0.9	<0.001	4.5 ± 0.5	5.7 ± 1.0	6.2 ± 0.8	6.7 ± 0.4	6.1 ± 0.9	<0.001
iPTH (pg/ml)	35.0(27.3–48.8)	404.9(194.7–1186.8)	<0.001	284.6(123.2–494.3)	2076.8(1306.2–2777.8)	1970.8(1370.5–3103.4)	1987.9(1248.1–3034.3)	1980.4(1343.4–2967.4)	<0.001
lniPTH	3.6 ± 0.4	6.0 ± 1.3	<0.001	5.5 ± 1.1	7.6 ± 0.5	7.6 ± 0.5	7.6 ± 0.5	7.6 ± 0.5	<0.001

(a) Data were mean ± standard deviation (SD), or numbers and percentages, or median (25th–75th percentile), as appropriate.

(b) Test of significance by Independent-Samples *t* test or Wilcoxon’s rank sum test for continuous variables and Chi-square test or Fisher’s exact test for categorical variables.

(c) *P*: controls versus stage 5 CKD patients; *P*^*′*^: No-PTX group versus PTX group.

(d) CKD, chronic kidney disease; PTX, parathyroidectomy; SHPT, secondary hyperparathyroidism; BMI, body mass index; BP, blood pressure; NA, not available; ACEI, angiotensin-converting enzyme inhibitors; ARB, angiotensin receptor blocker; HDL, high density lipoprotein; LDL, low density lipoprotein; TC, total cholesterol; ALP, alkaline phosphatase; iPTH, intact parathyroid hormone.

**Table 2:** Pearson correlation between lnleptin/BMI and clinical parameters in each group.

	Controls(n = 100)		Stage 5 CKD Patients(n = 209)		No-PTX Patients Group(n = 152)		PTX Patients Group(n = 57)	
	***r***	***P***	***r***	***P***	***r***	***P***	***r***	***P***
Age (years)	−0.050	0.621	0.107	0.125	0.092	0.262	0.157	0.244
Dialysis vintage (months)	NA	NA	−0.051	0.537	0.067	0.522	−0.126	0.350
Systolic BP (mmHg)	−0.130	0.198	−0.128	0.066	−0.101	0.215	−0.249	0.061
Diastolic BP (mmHg)	−0.169	0.092	−0.184	0.008	−0.140	0.085	−0.318	0.016
Hemoglobin (g/l)	−0.546	<0.001	0.117	0.091	0.172	0.034	−0.003	0.982
Hematocrit (%)	−0.530	<0.001	0.116	0.095	0.172	0.035	0.001	0.996
Glucose (mmol/l)	−0.175	0.081	0.134	0.054	0.155	0.056	0.024	0.859
Creatinine (μmol/l)	−0.477	<0.001	−0.201	0.003	−0.278	0.001	0.149	0.268
Urea (mmol/l)	−0.200	0.046	−0.167	0.016	−0.231	0.004	0.076	0.572
HDL cholesterol (mmol/l)	0.151	0.133	−0.209	0.002	−0.258	0.001	−0.090	0.507
LDL cholesterol (mmol/l)	−0.114	0.259	0.164	0.017	0.131	0.109	0.335	0.011
TC (mmol/l)	−0.073	0.470	0.115	0.098	0.113	0.167	0.118	0.383
Triglyceride (mmol/l)	−0.080	0.429	0.320	<0.001	0.308	<0.001	0.372	0.004
TC/HDL cholesterol	−0.147	0.144	0.288	<0.001	0.307	<0.001	0.223	0.096
Albumin (g/l)	−0.177	0.078	0.225	0.001	0.171	0.035	0.442	0.001
Calcium (mg/dl)	−0.001	0.990	0.231	0.001	0.255	0.002	0.323	0.014
Phosphorus (mg/dl)	0.369	<0.001	−0.006	0.927	−0.088	0.281	0.263	0.048
lnALP	0.081	0.425	−0.062	0.373	0.063	0.444	−0.287	0.031
lniPTH	0.157	0.120	−0.127	0.067	−0.143	0.079	−0.306	0.021

(a) Data were Pearson’s product moment correlation coefficient (*r*) and corresponding *P* values.

(b) BMI, body mass index; CKD, chronic kidney disease; PTX, parathyroidectomy; NA: not available; BP, blood pressure; HDL, high density lipoprotein; LDL, low density lipoprotein; TC, total cholesterol; iPTH, intact parathyroid hormone; ALP, alkaline phosphatase.

**Table 3:** Clinical parameters in follow-up PTX patients before and after PTX.

	**Successful PTX (n = 36)**			**Persistent SHPT (n = 4)**		
	**Before PTX**	**After PTX**	***P***	**Before PTX**	**After PTX**	***P***
Weight (kg)	56.8 ± 10.3	58.4 ± 10.8	0.025	57.3 ± 9.2	56.3 ± 11.4	0.572
BMI (kg/m^2^)	21.5 ± 3.1	21.9 ± 3.0	0.035	19.6 ± 2.5	19.3 ± 2.5	0.559
Laboratory Values						
Hemoglobin (g/l)	96.6 ± 19.5	113.4 ± 18.7	<0.001	109.0 ± 17.2	126.0 ± 21.4	0.153
Hematocrit (%)	30.1 ± 5.9	35.7 ± 6.0	<0.001	34.1 ± 5.0	39.2 ± 6.7	0.152
HDL cholesterol (mmol/l)	1.0 ± 0.3	1.2 ± 0.7	0.240	1.1 ± 0.2	1.0 ± 0.1	0.769
LDL cholesterol (mmol/l)	2.5 ± 0.6	2.7 ± 0.7	0.016	2.7 ± 0.5	2.3 ± 0.7	0.242
TC (mmol/l)	3.9 ± 0.8	4.4 ± 1.0	0.004	4.5 ± 1.1	4.4 ± 0.3	0.842
Triglyceride (mmol/l)	1.5 ± 0.9	1.8 ± 0.9	0.032	1.9 ± 1.3	2.1 ± 2.1	0.695
TC/HDL cholesterol	4.0 ± 1.2	4.1 ± 1.2	0.546	4.4 ± 1.2	4.5 ± 0.6	0.861
Albumin (g/l)	37.4 ± 3.3	43.7 ± 4.4	<0.001	37.0 ± 3.3	45.9 ± 3.8	0.014
Calcium (mg/dl)	10.1 ± 1.2	8.4 ± 1.2	<0.001	9.5 ± 0.9	8.9 ± 1.7	0.622
Phosphorus (mg/dl)	7.0 ± 2.2	3.6 ± 1.7	<0.001	7.0 ± 0.9	5.2 ± 1.9	0.216
lnALP	6.2 ± 0.8	5.1 ± 0.6	<0.001	6.7 ± 0.4	6.4 ± 0.1	0.342
lniPTH	7.6 ± 0.5	3.3 ± 1.9	<0.001	7.6 ± 0.5	6.8 ± 0.7	0.308
lnLeptin/BMI	5.5 ± 1.3	5.9 ± 1.2	<0.001	3.8 ± 1.3	3.9 ± 0.9	0.743

(a) Data were mean ± SD.

(b) Test of significance by a paired samples *t* test.

(c) *P*: before PTX versus after PTX.

(d) PTX, parathyroidectomy; SHPT, secondary hyperparathyroidism; BMI: body mass index; HDL, high density lipoprotein; LDL, low density lipoprotein; TC, total cholesterol; iPTH, intact parathyroid hormone.

(e) The data of postoperative weight and lnleptin/BMI in successful PTX group was incomplete (n = 35).
