# Supplementary material for: Association of Increased Serum Leptin with Ameliorated Anemia and Malnutrition in Stage 5 Chronic Kidney Disease Patients after Parathyroidectomy
Source: Sci Rep. 2016 Jun 16;6:27918. doi: 10.1038/srep27918 (PMC4910047; doi:10.1038/srep27918)
Supplement: Supplementary Information [file srep27918-s1.pdf]

Association of Increased Serum Leptin with Ameliorated Anemia and Malnutrition in Stage 5 Chronic Kidney Disease Patients after Parathyroidectomy

Authors

Yao Jiang,[1,6] Jingjing Zhang,[1,6] Yanggang Yuan,[1] Xiaoming Zha,[2] Changying Xing,[1] Chong Shen,[3] Zhixiang Shen,[4] Chao Qin,[5] Ming Zeng,[1] Guang Yang,[1] Huijuan Mao,[1] Bo Zhang,[1] Xiangbao Yu,[1] Bin Sun,[1] Chun Ouyang,[1] Xueqiang Xu,[1] Yifei Ge,[1] Jing Wang,[1] Lina Zhang,[1] Chen Cheng,[1] Caixia Yin,[1] Jing Zhang,[1] Huimin Chen,[1] Haoyang Ma,[1] and Ningning Wang[1*].

[1]Department of Nephrology, The First Affiliated Hospital with Nanjing Medical University, Nanjing, Jiangsu Province, 210029, China.

[2]Department of General Surgery, The First Affiliated Hospital with Nanjing Medical University, Nanjing, Jiangsu Province, 210029, China.

[3]Department of Epidemiology and Biostatistics, School of Public Health, Nanjing Medical University, Nanjing, Jiangsu Province, 210029, China.

[4]Department of Nephrology, Jiangsu Province Geriatric Hospital, Nanjing, Jiangsu Province, 210024, China.

[5]Department of Urology, The First Affiliated Hospital with Nanjing Medical University, Nanjing, Jiangsu Province, 210029, China.

[6]These authors contributed equally to this work.

[*]Corresponding author.

Figure S1. Higher proportion of low BMI in CKD patients with lower lnleptin/BMI.

A. The proportion of BMI <23 kg/m^2 in CKD patients was higher than those in controls (64.6% *vs* 40.0%, *P*<0.001).

B. CKD patients in BMI <23 kg/m^2 group had lower circulating lnleptin/BMI levels than those in BMI ≥23 kg/m^2 group (5.2±1.3 *vs* 5.9±1.4, *P*=0.001). Compared to controls, CKD patients had higher lnleptin/BMI in BMI ≥23 kg/m^2 group (5.3±0.8 *vs* 5.9±1.4, *P*=0.002). In BMI <23 kg/m^2 group, lnleptin/BMI levels in CKD patients were lower than those in controls although without statistical significance (5.4±0.7 *vs* 5.2±1.3, *P*=0.131).

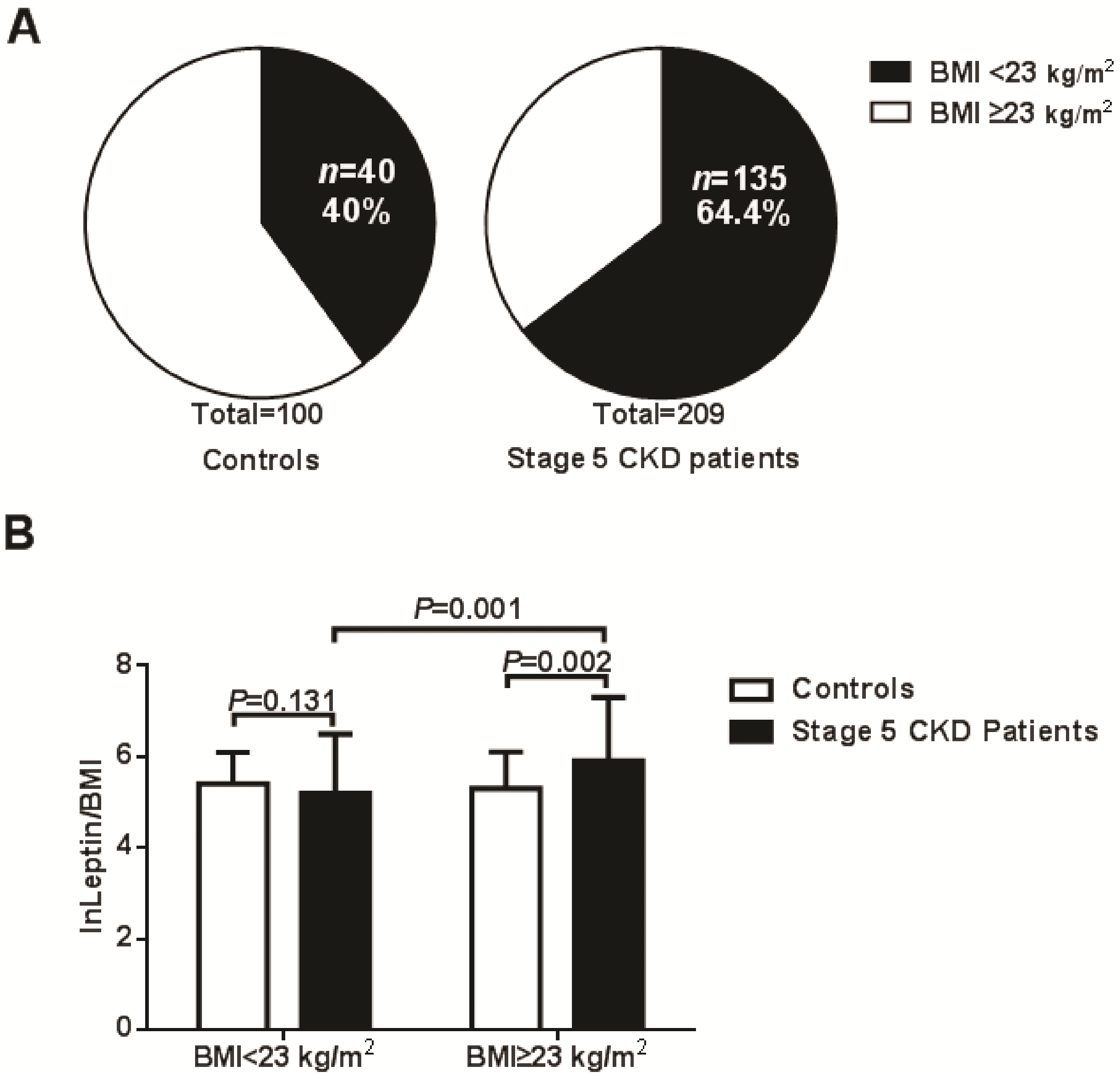
A
BMI <23 kg/m2
BMI ≥23 kg/m2
n=40
40%
n=135
64.4%
Total=100
Controls
Total=209
Stage 5 CKD patients
B
P=0.001
P=0.002
P=0.131
Controls
Stage 5 CKD Patients
lnLeptin/BMI
0
2
4
6
8
BMI<23 kg/m2
BMI≥23 kg/m2

Table S1, Follow-up clinical parameters in successful PTX patients divided by baseline BMI.

	BMI≥23 kg/m^2 (n=13)			BMI<23 kg/m^2 (n=23)		
	Before PTX	After PTX	*P*	Before PTX	After PTX	*P*
Weight(kg)	67.5±8.4	67.8±10.5	0.767	51.7±6.3	53.5±7.0	0.004
BMI(kg/m^2)	25.2±1.4	25.3±2.0	0.862	19.5±1.5	20.2±1.7	0.004
Laboratory Values						
Hemoglobin(g/l)	91.5±21.3	109.4±16.8	0.002	99.4±18.3	115.6±19.7	0.002
Hematocrit(%)	28.4±6.8	33.9±5.3	0.003	31.0±5.2	36.7±6.3	<0.001
TC(mmol/l)	3.7±0.9	4.3±0.9	0.087	4.0±0.8	4.4±1.0	0.024
Triglyceride(mmol/l)	1.8±1.3	2.2±1.2	0.294	1.3±0.5	1.6±0.6	0.029
HDL cholesterol(mmol/l)	0.9±0.2	1.3±1.2	0.267	1.1±0.3	1.1±0.3	0.771
LDL cholesterol(mmol/l)	2.6±0.6	2.7±0.7	0.534	2.4±0.5	2.7±0.8	0.017
TC/HDL cholesterol	4.1±1.2	4.1±1.4	0.925	3.9±1.2	4.1±1.1	0.298
Albumin(g/l)	38.1±3.0	44.2±3.6	<0.001	37.0±3.4	43.4±4.8	<0.001
Calcium(mg/dl)	10.3±1.7	8.8±1.3	0.012	10.0±0.8	8.2±1.1	<0.001
Phosphorus(mg/dl)	7.4±1.9	3.9±1.8	<0.001	6.9±2.3	3.5±1.7	<0.001
lnALP	5.9±0.7	4.9±0.5	<0.001	6.4±0.8	5.2±0.7	<0.001
lniPTH	7.3±0.5	3.3±1.9	<0.001	7.7±0.4	3.3±2.0	<0.001
lnLeptin/BMI	6.1±1.2	6.6±1.3	0.005	5.1±1.2	5.6±1.1	0.017

Data were mean ±standard deviation (SD);
Test of significance by a paired samples *t* test;
P: before PTX versus after PTX;
PTX, parathyroidectomy; BMI, body mass index; TC, total cholesterol; HDL, high density lipoprotein; LDL, low density lipoprotein; ALP, alkaline phosphatase; iPTH, intact parathyroid hormone;
The data of postoperative weight and lnleptin/BMI in BMI>23 kg/m^2 group were incomplete (n=12).

Supplemental Experimental Procedures

Protocols

It has been previously shown that leptin release is a pulsatile manner, and modulated by additional short-term signals, i.e., the levels increase acutely after a meal, and are suppressed with fasting[1]. Leptin concentrations also follow a diurnal rhythm which is highest between midnight and early morning and lowest in midafternoon[2]. In our study at enrollment, venous whole blood samples were drawn in the morning from the participants with an overnight fast. For hemodialysis patients, blood samples were collected before dialysis. Clinical characteristics, medical history, and use of anti-hypertension medications were strictly recorded.

Analysis of Laboratory Values

Routine blood tests were performed using the LH-750 Hematology Analyzer from Beckman Coulter, Inc. Fullerton, CA. Biochemical indices were measured using an Automatic Biochemical Analyzer named AU5400 from Olympus Corporation, Tokyo, Japan. Serum iPTH levels were measured using a UniCel DxI800 Access Immunoassay System from Beckman Coulter, Inc. Fullerton, CA. Human serum leptin levels were measured using the Human Leptin Quantikine ELISA Kit from R&D Systems, Minneapolis, USA. The inter-assay variation coefficient of human leptin ELISA kit ranged from 3.0% to 3.3%. The lower sensitivity limit of the assay was 7.8 pg/ml.

Reagents and Chemicals

Dulbecco's Modified Eagle Medium/high glucose supplemented (DMEM) and fetal bovine serum (FBS) were purchased from Gibco, New York, USA. 3-Isobutyl-1-methylxanthine (IBMX), dexamethasone, insulin, rat parathyroid hormone (1-34) and oil red O were obtained from Sigma-Aldrich, Louis, USA. Anti-leptin antibody was obtained from Abcam, Cambridge, UK. Antibody against Akt, phosphate-Akt (Ser473) and Glyceraldehyde-3-phosphate dehydrogenase (GAPDH) were purchased from Cell Signaling Technology, Boston, USA. LY294002 (phosphatidylinositol 3-kinase [PI3K] inhibitor) was from MedChem Express, New Jersey, USA. ECL Detection Systems were from Amersham, Buckinghamshire, UK. Mouse Leptin Quantikine ELISA Kits used for measurement of leptin in cell culture media were from R&D Systems, Minneapolis, USA. The inter-assay variation coefficient of mouse leptin ELISA kit ranged from 3.3% to 4.3%. The lower sensitivity limit of the assay was 22 pg/ml.

Western Blot

The immunoblotting procedure was previously described[3]. Briefly, 30 μg protein extracts were separated by acrylamide electrophoresis and transferred to polyvinylidene fluoride membranes. After 1 hour incubation at room temperature in 5% dry milk powder, the membranes were immunoblotted with primary antibodies against Leptin (1:2000), phosphate-Akt (1:1000), Akt (1:1000) or GAPDH (1:1000), and followed by the addition of HRP-labeled secondary antibodies. The blots were visualized with Amersham ECL Detection Systems. Quantitative analysis of immunoblotting images was performed using Image Lab software from Bio-Rad Laboratories, California, USA.

Clinical Definition

Successful PTX was defined as normalization of serum calcium, serum phosphorus, alkaline phosphatase, and maintenance of no more than two to three times serum iPTH compared with normal (ranging from 10–88 pg/ml). The serum iPTH levels detected during the first postoperative week >300 pg/ml were considered indicative of persistent SHPT[4]. In these cases, SHPT was partially but not completely corrected. Patients with persistent SHPT after PTX were commonly considered to have supernumerary/ectopic parathyroid glands and remain one or two parathyroid glands while most of them had being moved during surgery[5]. The diagnosis of anemia was made at the hemoglobin concentrations: <13.5 g/dl in adult males, <12.0 g/dl in adult females[6].

References

1 Dallongeville, J. *et al.* Short term response of circulating leptin to feeding and fasting in man: influence of circadian cycle. *Int J Obes Relat Metab Disord* **22**, 728-733 (1998).

2 Sinha, M. K. *et al.* Nocturnal rise of leptin in lean, obese, and non-insulin-dependent diabetes mellitus subjects. *J Clin Invest* **97**, 1344-1347, doi:10.1172/JCI118551 (1996).

3 Jia, Z. *et al.* Amelioration of cisplatin nephrotoxicity by genetic or pharmacologic blockade of prostaglandin synthesis. *Kidney Int* **79**, 77-88, doi:10.1038/ki.2010.331 (2011).

4 Kara, M. *et al.* Evaluation of intraoperative parathormone measurement for predicting successful surgery in patients undergoing subtotal/total parathyroidectomy due to secondary hyperparathyroidism. *Laryngoscope* **120**, 1538-1544, doi:10.1002/lary.21023 (2010).

5 Schneider, R. *et al.* Frequency of ectopic and supernumerary intrathymic parathyroid glands in patients with renal hyperparathyroidism: analysis of 461 patients undergoing initial parathyroidectomy with bilateral cervical thymectomy. *World J Surg* **35**, 1260-1265, doi:10.1007/s00268-011-1079-6 (2011).

6 KDOQI & NationalKidneyFoundation. KDOQI Clinical Practice Guidelines and Clinical Practice Recommendations for Anemia in Chronic Kidney Disease. *Am J Kidney Dis* **47**, S11-145, doi:10.1053/j.ajkd.2006.03.010 (2006).
